# First person – Salil Sukumaran

**DOI:** 10.1242/dmm.049929

**Published:** 2022-10-18

## Abstract

First Person is a series of interviews with the first authors of a selection of papers published in Disease Models & Mechanisms, helping researchers promote themselves alongside their papers. Salil Sukumaran is first author on ‘ Abnormalities in migration of neural precursor cells in familial bipolar disorder’, published in DMM. Salil is a senior scientific officer in the lab of Biju Viswanath at the Department of Psychiatry, National Institute of Mental Health and Neurosciences, Bangalore, India, investigating the role of cellular migration in bipolar disorder.


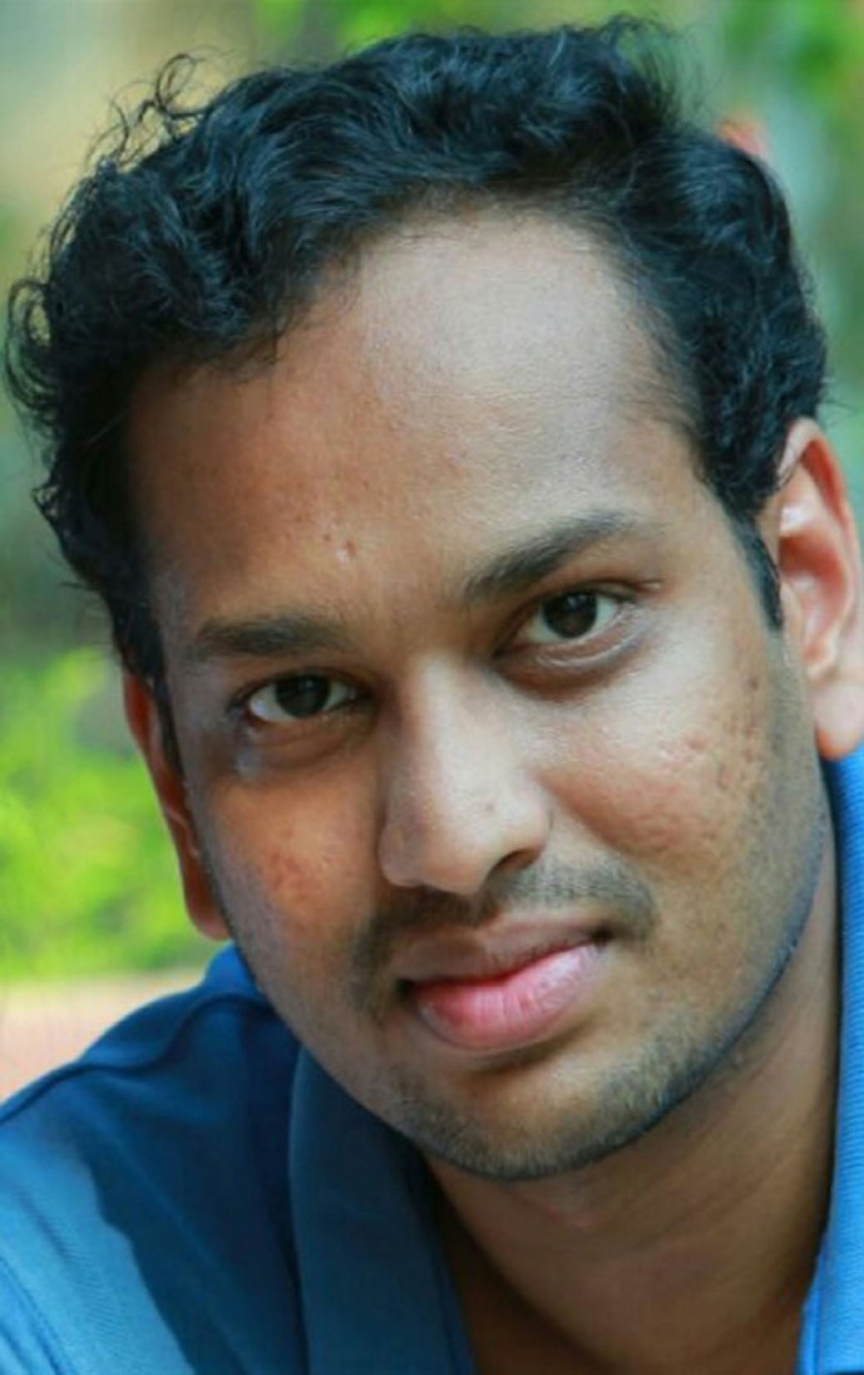


**Salil Sukumaran**

**How would you explain the main findings of your paper to non-scientific family and friends?**

In this paper, we looked at brain development in bipolar disorder. Using cells called neural precursors, which give rise to many of the cells that make up the adult brain, we found that migration or movement of these cells is highly irregular in this disease. Considering the size of our brains, one can imagine the huge distances that these precursor cells would have to travel during the development of the brain. It is the equivalent of a human being having to walk thousands of kilometers. Only these cells have to do that without any GPS or Google Maps! Normal brain development relies on the ability of these cells to consistently and accurately read the signals in their environment, which guide them as to when they should move straight, when to pause, when to turn, and so on, until they get to their final destination in the growing brain. If these cells misread these signals, brain development can go very wrong, leading to structural changes in the brain. What we report in this paper suggests that this might be part of the problem in bipolar disorder.

**What are the potential implications of these results for your field of research?**

Psychiatric diseases like bipolar disorder are highly complex. Any one technique or model system to study these diseases will give us one piece of the puzzle, one clue. What we really ought to do is to put different kinds of clues together in order to get a more comprehensive picture of what goes wrong in the brain. This is precisely the reason that the ADBS (Accelerator program for Discovery in Brain disorders using Stem cells) consortium was created, under which the present study was funded. I designed and conducted the experiments using precursor cells. My colleagues conducted and analysed MRIs, while other colleagues interpreted gene expression patterns. For this paper, we put all these clues together, which told us that (1) there are structural differences in brains of patients, picked up on the MRI; (2) there are gene expression changes linked to migration; and (3) the kind of migration that we see in cells from patients is abnormal. Taking these findings together with the results from a previous paper from the consortium, which showed there are also changes (variations) in many gene sequences that code for different proteins involved in the migration process, we are starting to piece together what goes wrong during brain development in bipolar disorder.

“Cells derived from patients […] offer the option of studying cellular responses in the context of the entire genetic repertoire of the individuals.”

**What are the main advantages and drawbacks of the experimental system you have used as it relates to the disease you are investigating?**

Cells derived from patients, as in this case, offer the option of studying cellular responses in the context of the entire genetic repertoire of the individuals. That is one of the major advantages. Every model system will have its limitations. Cells from patients, even with limitations, are currently the best system to probe the biological basis of psychiatric diseases. An obvious limitation is that a developing brain is a lot more complex, consisting of many cell types other than neural precursors. How cells behave is also a response to their neighboring cells. We are missing that key component in the two-dimensional system that we used for this study. To some extent, three-dimensional (3D) organoids can help resolve those limitations. These are what we are presently using for our next steps in studying bipolar disorder.

**Figure:**
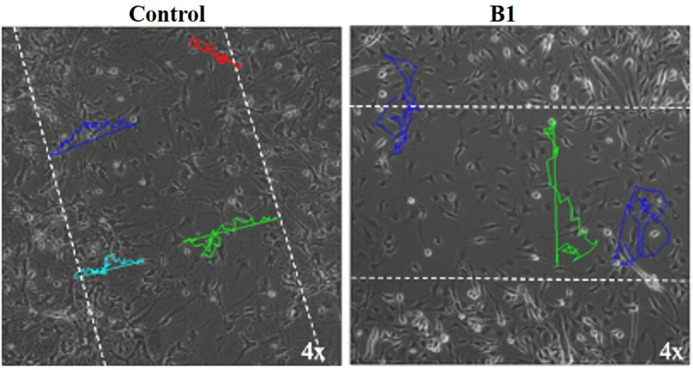
**Migratory path of neural precursor cells of a control and bipolar disorder patient assessed over a 15-h period.** Compared to cells from the healthy control, cells from the patient move in a highly irregular and random manner.

**What has surprised you the most while conducting your research?**

The extent of abnormal migration patterns that we noticed in the very first patient-derived cell line studied (B1 in this paper) – that was surprising. To some extent, we expected to find some differences in cell migration, but not to the extent as these cells showed.

**What do you think is the most significant challenge impacting your research at this time and how will this be addressed over the next 10 years?**

The translatability of findings from basic research to the clinic, to help patients – that is a major challenge. I hope that basic research findings like this can move us forward to the point of harnessing this knowledge for bettering therapeutic options for patients.

**What changes do you think could improve the professional lives of scientists?**

Being able to network with one's peers is of utmost importance in advancing your career. Thus, funding opportunities for scientists from low- and middle-income countries to be able to attend meetings, especially international conferences, are very crucial in my view. Such opportunities are also very limited currently, which adds to our challenges.

**What's next for you?**

Firstly, I want to look at the behavior of these precursor cells in a 3D model system (organoids). That will tell us more about how the abnormalities that we report in this paper might shape brain development differently. Secondly, I also want to examine the same response (migration of neural precursors) in cells from more bipolar disorder patients. Finally, I would also like to examine how abnormal migration fits in with other processes in brain development, to ultimately lead to disease in the adult brain.

## References

[DMM049929C1] Sukumaran, S. K., Paul, P., Guttal, V., Holla, B., Vemula, A., Bhatt, H., Bisht, P., Mathew, K., Nadella, R. K., Varghese, A. M. et al. (2022). Abnormalities in migration of neural precursor cells in familial bipolar disorder. *Dis. Model. Mech.* 15, dmm049526. 10.1242/dmm.049526 36239094 PMC9612872

